# Prospective Keratograph 5 M Assessment Following Intradermal Botulinum Toxin Type A for Seborrhea and Enlarged Facial Pores

**DOI:** 10.1111/jocd.71073

**Published:** 2026-07-14

**Authors:** L. A. Dominguez‐Salgado, R. Ramirez‐Hernandez, A. Nava‐Castañeda

**Affiliations:** ^1^ Oculoplastic Department Instituto de Oftalmologia Fundacion Conde de Valenciana Mexico City Mexico

**Keywords:** botulinum toxins type A, intradermal injections, seborrhea, sebum, skin aging

## Abstract

**Background:**

Seborrhea and enlarged facial pores are common cosmetic concerns associated with increased sebum production and impaired skin quality. Conventional treatments often have variable efficacy and may cause adverse effects. Intradermal botulinum toxin type A has emerged as a minimally invasive alternative.

**Aims:**

To explore the clinical outcomes associated with intradermal botulinum toxin type A treatment for seborrhea and enlarged facial pores using objective and patient‐reported measures.

**Methods:**

A prospective study involving 30 patients with oily skin and enlarged facial pores was conducted. All participants received intradermal botulinum toxin type A injections into both cheeks. Outcomes were assessed at baseline and 6 weeks post‐treatment. Sebum production was measured using oil‐absorbing sheets (mm^2^), and pore size was measured using an Oculus Keratograph 5 M. Patient‐reported outcomes were evaluated with the FACE‐Q, and skin quality was evaluated with the SASS‐Q. Statistical analysis was performed using paired tests with significance set at *p* < 0.05.

**Results:**

Sebum production significantly decreased on both sides of the face (right: *p* = 0.0016; left: *p* = 0.0011). The mean values decreased from 291.9 to 191.6 mm^**2**^ (right) and from 290.5 to 187.1 mm^2^ (left). Pore size also showed a reduction (*p* < 0.0001), decreasing from 531.3 to 235.3 μm (right) and from 554 to 241.3 μm (left). The FACE‐Q scores improved from 50.87 to 70.07, whereas the SASS‐Q scores decreased from 2.1 to 1.47 (both *p* < 0.0001).

**Conclusions:**

Improvements in sebum production, facial pore size, and patient‐reported outcomes were observed following intradermal botulinum toxin type A treatment.

**Trail Registration:**

ClinicalTrials.gov identifier: NCT07387536

## 1 Introduction

Seborrhea and enlarged facial pores are common dermatological and cosmetic concerns associated with increased sebaceous gland activity and altered skin texture [1, 2]. Excessive sebum production contributes to a shiny skin appearance, acne development, and pore dilation, which may negatively impact patients' quality of life and self‐perception [1].

Facial pores represent the openings of pilosebaceous units and are influenced by multiple factors, including sebum production, age, hormonal status, genetic predisposition, exposure to UV light, and ethnicity [2]. Increased sebaceous activity and reduced skin elasticity contribute to pore enlargement, particularly in the central facial region [3]. Conventional treatments for seborrhea and enlarged pores include topical retinoids, hormonal therapy, isotretinoin, and energy‐based devices; however, these approaches may have variable efficacy and are often associated with adverse effects [2, 4].

Botulinum toxin type A (BoNT‐A), widely used for cosmetic and therapeutic indications, has recently attracted attention as a potential treatment for seborrhea and enlarged pores [4]. Intradermal administration of BoNT‐A, commonly referred to as “microbotox,” involves the injection of diluted toxin into the superficial dermis, targeting sebaceous glands and superficial muscle fibers [4]. This technique allows the modulation of skin quality without significant muscle paralysis.

The proposed mechanism of action involves inhibition of acetylcholine release, which plays a role in sebocyte differentiation and sebaceous gland activity [5]. Sebaceous glands express nicotinic acetylcholine receptors, and blockade of cholinergic signaling may reduce sebum production [6, 7]. Additionally, BoNT‐A may affect the arrector pili muscle and local microenvironment, contributing to reduced pore size and improved skin texture [3, 4].

Although previous studies have demonstrated promising effects of intradermal botulinum toxin type A on sebum production and pore appearance, additional prospective data remain valuable for assessing reproducibility across different clinical settings and patient populations [2, 6, 7, 8]. Therefore, this study aimed to evaluate changes in sebum production and facial pore size following intradermal botulinum toxin type A treatment using both objective measurements and patient‐reported outcomes while providing additional prospective data from a Hispanic cohort.

## 2 Methods

A prospective, longitudinal, before‐and‐after study was conducted to evaluate the effects of intradermal botulinum toxin type A on sebum production and facial pore size. Given the exploratory nature of the study, a formal a priori sample size calculation was not performed. Instead, the sample size was determined according to the recommendations of Browne and Kieser and Wassmer for preliminary prospective studies, which suggest including 20–40 participants in exploratory clinical investigations. Accordingly, 30 participants were enrolled. Patients aged 18–35 years with oily skin and enlarged facial pores who attended the Oculoplastics Department were recruited. The inclusion criteria included a clinical diagnosis of oily skin, the presence of enlarged facial pores, and signed informed consent. The exclusion criteria were pregnancy or lactation, neuromuscular disorders, previous adverse reactions to botulinum toxin, recent aesthetic treatments (laser, chemical peeling, or botulinum toxin within the previous 6 months), use of isotretinoin, active skin infection in the treatment area, or incomplete questionnaires. A 500‐unit vial of botulinum toxin type A (Dysport, Ipsen, Paris, France) was reconstituted with 7 mL of sterile saline. After facial cleansing with isopropyl alcohol, five injection points were marked on each cheek in a standardized grid pattern. Three units were injected intradermally at each point using a 30‐gauge needle inserted at approximately 45°, ensuring superficial placement with formation of a small wheal, for a total dose of 15 units per cheek. Sebum production was assessed semiquantitatively using oil‐absorbing sheets. One sheet was applied to each cheek for 10 min, and the translucent area produced by sebum absorption was measured in mm^2^. Assessments were performed at baseline and at the 6‐week follow‐up.

Facial pore size was assessed using the OCULUS Keratograph 5 M (Oculus Optikgeräte GmbH, Wetzlar, Germany). High‐resolution color images of the malar region were obtained under standardized imaging conditions. For each cheek, the selected malar region was divided into four quadrants, and three representative pores were identified within each quadrant, yielding a total of 12 pores per cheek. The diameter of each pore was measured, and the mean diameter of the 12 pores was used for statistical analysis. At the 6‐week follow‐up, the same anatomical regions and corresponding pores were re‐identified using identical imaging settings and patient positioning before repeat measurements were obtained, ensuring measurement reproducibility between visits. All image analyses were performed by the same examiner. Patient‐reported outcomes were evaluated using the FACE‐Q questionnaire, and skin quality was assessed using the SASS‐Q scale. Standardized clinical photographs, Keratograph measurements, sebum assessments, and questionnaires were obtained at baseline and repeated 6 weeks after treatment.

Quantitative variables are expressed as the mean ± standard deviation or median and interquartile range, depending on the data distribution. Normality was assessed using the Shapiro–Wilk test, and paired comparisons were performed using Student's *t*‐test or the Wilcoxon signed‐rank test, as appropriate. A *p* value < 0.05 was considered to indicate statistical significance. Statistical analyses were performed using R software version 4.0.2. This study was approved by the Institutional Ethics Committee at the Ophthalmology Institute Fundación Conde de Valenciana (Approval Number: CEI‐2024/07/01) and adhered to the principles of the Declaration of Helsinki. Written informed consent was obtained from all participants before enrollment.

## 3 Results

**FIGURE 1:**
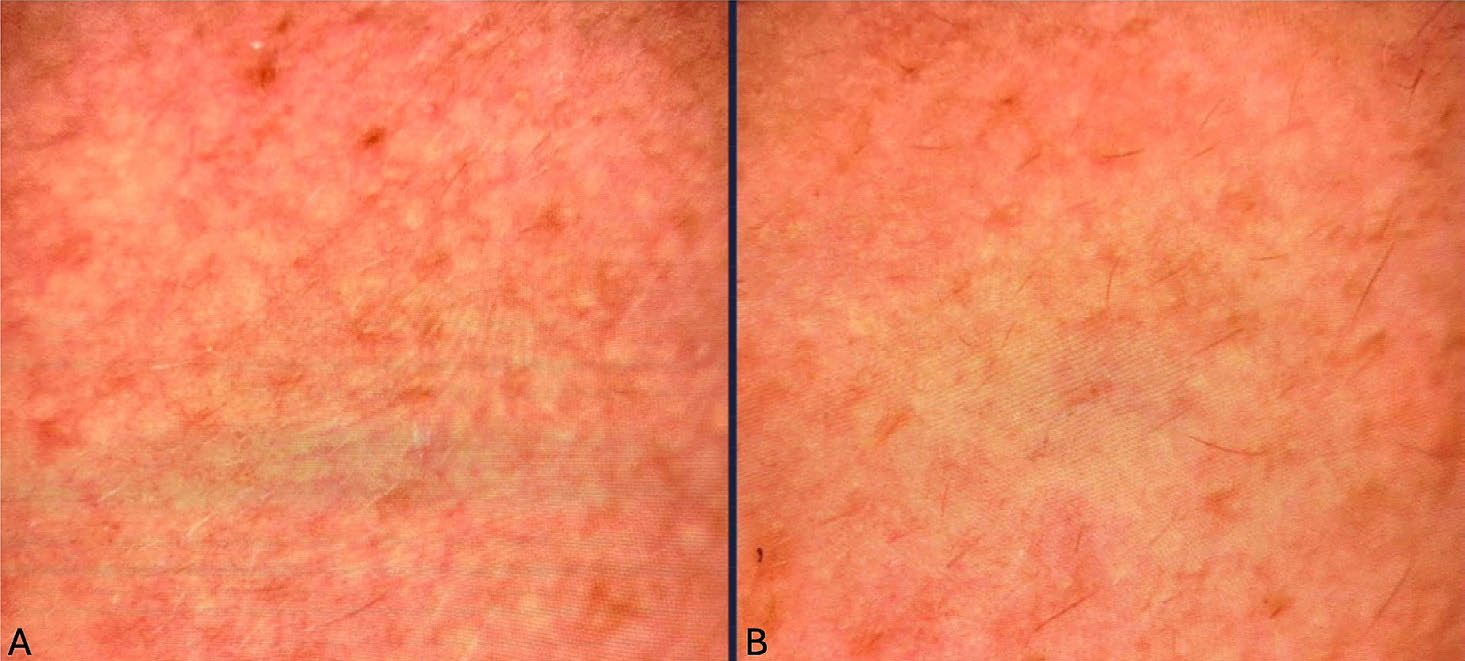
Representative clinical images showing facial skin before and 6 weeks after intradermal botulinum toxin treatment. (A) Baseline image prior to treatment. (B) Image obtained 6 weeks after treatment, demonstrating a visible reduction in pore size and improvement in skin texture. Images were obtained using an Oculus Keratograph 5 M.

**FIGURE 2:**
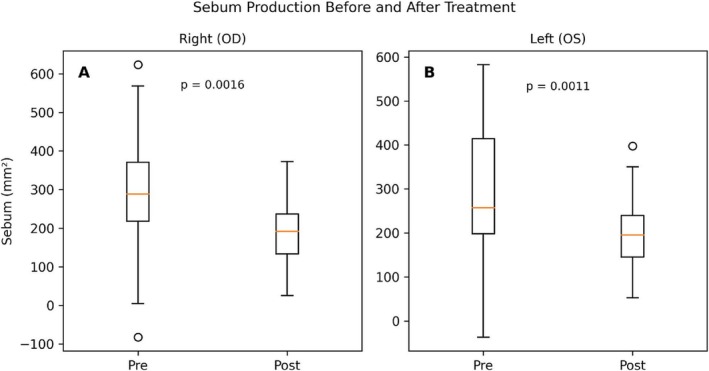
Boxplots demonstrating a significant reduction in sebum production (mm^2^) following intradermal botulinum toxin treatment on both the right (OD) and left (OS) sides of the face at 6 weeks (right: *p* = 0.0016; left: *p* = 0.0011). Boxes indicate the interquartile range, with median values and full data dispersion represented.

**FIGURE 3:**
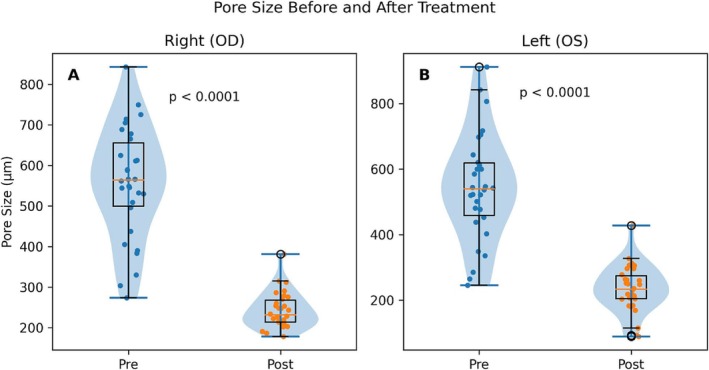
Distribution of facial pore size (μm) before and after intradermal botulinum toxin treatment, combining violin plots, boxplots, and individual data points. A marked and statistically significant reduction in pore size was observed bilaterally at 6 weeks (*p* < 0.0001 for both sides), demonstrating a marked reduction in pore size following treatment.

**FIGURE 4:**
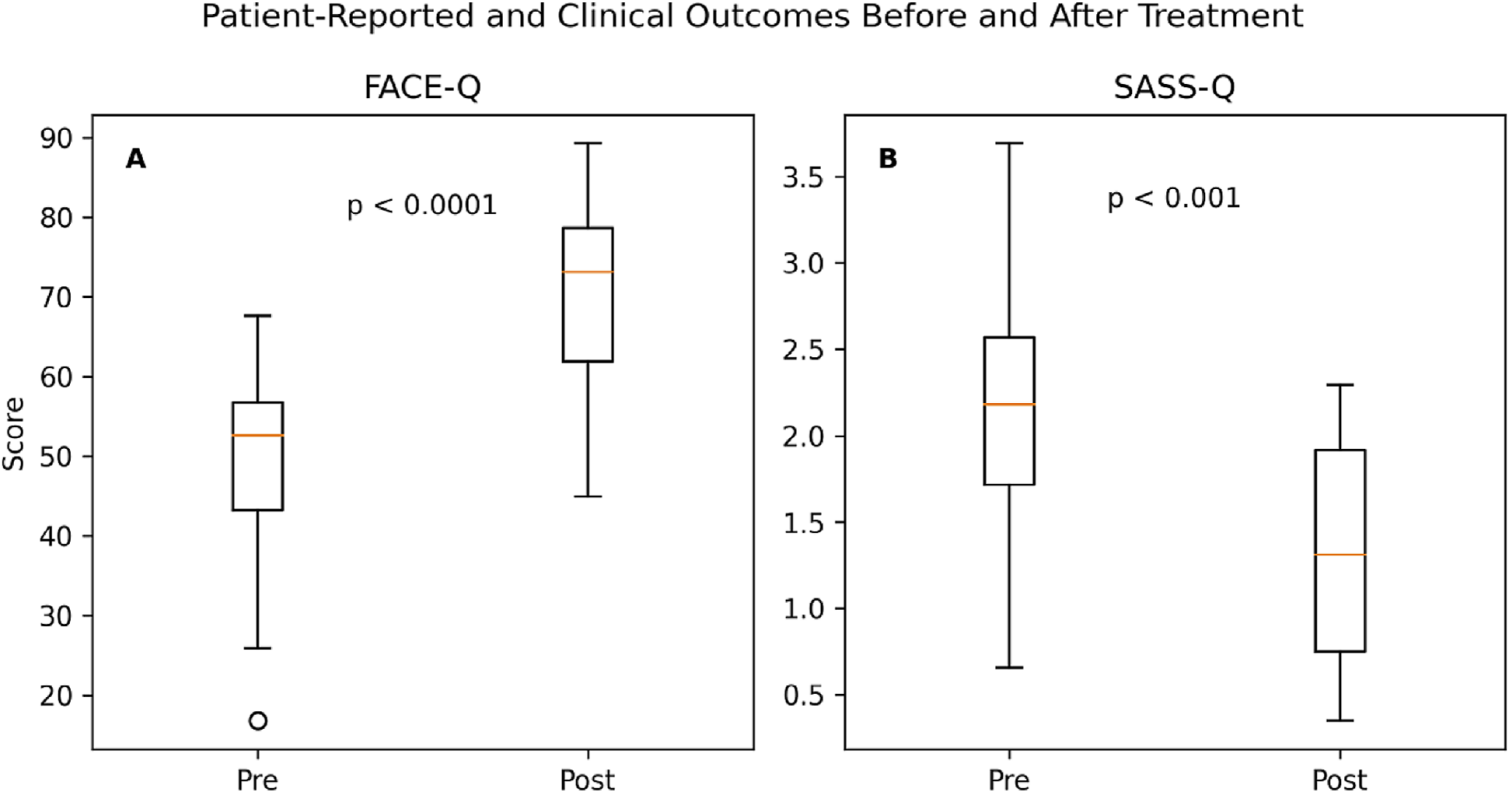
Boxplot comparison of patient‐reported and clinician‐assessed outcomes before and after intradermal botulinum toxin treatment. (A) FACE‐Q scores significantly increased following treatment, indicating improved patient satisfaction (*p* < 0.0001). (B) SASS‐Q scores significantly decreased, reflecting improvements in skin texture and pore characteristics (*p* < 0.001). Boxes represent the interquartile range (IQR), horizontal lines indicate the median, and whiskers represent the data range.

A total of 33 patients were initially recruited; however, 3 were excluded because of incomplete follow‐up, resulting in a final sample of 30 participants. Among these patients, 70% (*n* = 21) were female and 30% (*n* = 9) were male, with a median age of 27 years (IQR: 2.5 years). Visible improvements in skin texture and pore appearance were observed following treatment (Figure 1). Sebum production decreased after treatment on both sides of the face, with mean values decreasing from 291.9 ± 163.69 mm^2^ to 191.6 ± 80.39 mm^2^ on the right side (mean reduction: 100.3 mm^2^; *p* = 0.0016) and from 290.5 ± 171.92 mm^2^ to 187.1 ± 93.22 mm^2^ on the left side (mean reduction: 103.4 mm^2^; *p* = 0.0011) (Figure 2). Facial pore size also decreased following treatment (*p* < 0.0001 for both sides), with mean values decreasing from 531.3 ± 130.54 μm to 235.3 ± 60.78 μm on the right side (mean reduction: 296 μm) and from 554 ± 144.90 μm to 241.3 ± 77.71 μm on the left side (mean reduction: 312.7 μm) (Figure 3). Patient‐reported outcomes also improved following treatment, with FACE‐Q scores increasing from a mean of 50.87 ± 10.66 to 70.07 ± 9.30 (mean increase: 19.2 points; *p* < 0.0001). Similarly, the SASS‐Q score decreased from 2.1 ± 0.76 to 1.47 ± 0.57 (mean reduction: 0.63 points; *p* < 0.0001) (Figure 4). No serious adverse events or clinically significant treatment‐related complications were observed during the follow‐up period. Adverse events assessed during follow‐up included pain, erythema, edema, bruising, infection, asymmetry, muscle weakness, and other treatment‐related complications. Mild transient injection‐site discomfort and erythema, when present, were self‐limiting.

## 4 Discussion

These findings are consistent with those of previous reports suggesting that intradermal microinjections of botulinum toxin type A (BoNT‐A) may reduce sebum production and improve facial pore appearance [4, 6, 7, 8]. In this prospective observational cohort, improvements were observed across both objective measurements and patient‐reported outcomes following treatment. The observed reductions in sebum production and pore size are in line with previously proposed mechanisms involving cholinergic modulation of sebaceous gland activity and perifollicular dynamics. By interfering with acetylcholine‐mediated pathways, BoNT‐A may influence sebocyte differentiation and lipid synthesis, thereby contributing to reduced sebum production [2]. Similarly, improvement in facial pore appearance may reflect combined effects involving decreased sebaceous secretion and perifollicular neuromodulation. Importantly, improvements in FACE‐Q and SASS‐Q scores support the clinical relevance of these findings, suggesting that objective changes were accompanied by meaningful patient‐perceived benefits. The consistency between objective and patient‐reported outcomes further supports the potential applicability of microtoxin in aesthetic practice.

Previous studies have also demonstrated favorable outcomes following intradermal botulinum toxin treatment for seborrhea and enlarged facial pores. Sayed et al. [6] reported significant reductions in sebum production and pore size in a split‐face controlled study, with sustained improvement lasting up to 4 months. Similarly, Ahmed El Attar and Nofal described microbotox as a promising therapeutic approach for enlarged facial pores, demonstrating significant clinical improvement in facial skin texture and pore appearance. Salem et al. [7] further reported greater efficacy and longer‐lasting results with intradermal microbotox injection compared with topical application following microneedling. The results of the present study support these previous observations while contributing additional prospective data using objective Keratograph‐based assessment and patient‐reported outcome measures in a Hispanic cohort.

Nevertheless, several limitations should be acknowledged. The absence of a control group precludes definitive causal inference and does not allow differentiation between treatment‐related effects, placebo response, or natural variability. Additionally, the relatively small sample size and short follow‐up period limit generalizability and preclude long‐term assessment. Multiple outcomes were evaluated without formal adjustment for multiple comparisons, which may increase the risk of type I error. Bilateral facial measurements were also analyzed separately without adjustment for within‐subject correlation. Therefore, these findings should be interpreted as supportive prospective observational data rather than definitive evidence of efficacy.

## 5 Conclusion

Intradermal microinjections of botulinum toxin type A were associated with improvements in sebum production, facial pore size, and patient‐reported outcomes. Although these findings support a potential role for microtoxin in the management of seborrhea and enlarged facial pores, the absence of a control group, limited sample size, and short follow‐up period warrant cautious interpretation. Further randomized controlled studies with larger cohorts and longer follow‐up periods are needed to confirm treatment efficacy and durability.

## Author Contributions

All the authors have read and approved the final manuscript. L.A.D.‐S. and A.N.‐C. Performed the research. L.A.D.‐S., and A.N.‐C. designed the research study. L.A.D.‐S. and A.N.‐C. analyzed the data. L.A.D.‐S., R.R.‐H. and A.N.‐C. wrote the paper.

## Funding

The authors have nothing to report.

## Ethics Statement

The authors confirm that the ethical policies of the journal, as noted on the journal's author guidelines page, have been adhered to and the appropriate ethical review committee approval has been received. Institutional Ethics Committee Board (Registration Number: CEI‐2025/05/05). Consent has been obtained from participants for the publication of photographs.

## Conflicts of Interest

The authors declare no conflicts of interest.

## Data Availability

The data that support the findings of this study are available on request from the corresponding author. The data are not publicly available due to privacy or ethical restrictions.
